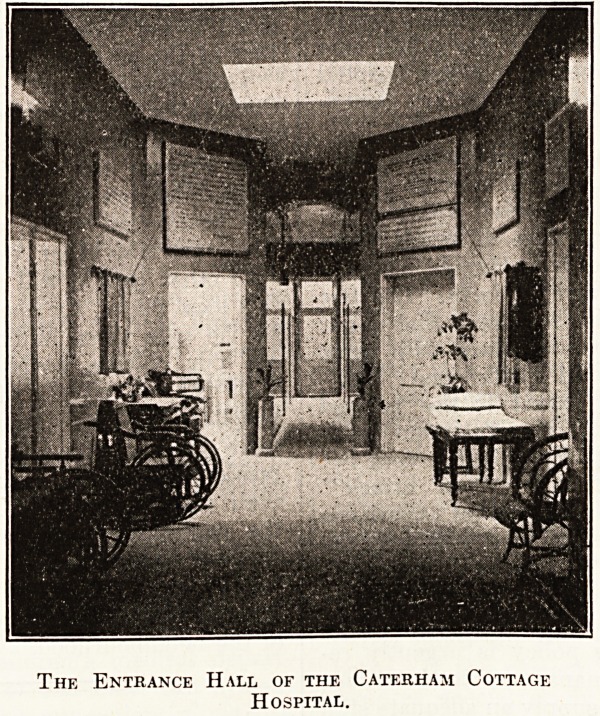# Reports on Hospitals of the United Kingdom

**Published:** 1914-07-18

**Authors:** Henry Burdett


### REPORTS ON

### Hospitals of the United Kingdom.

By SIR HENRY BURDETT, K.C.B., K.C.V.O.

SERIES III.

### CATERHAM COTTAGE HOSPITAL.

This hospital was established in 1875 in memory of a lady who spent her life in doing good works for others and whose memory is fittingly commemorated by a printed record which is suspended in the entrance hall, together with other inscriptions to benefactors of, and workers for, this interesting hospital. It was rebuilt in 1903 and has had some recent additions, including a dining room for the nursing staff. We congratulate the architect on the way in which he has carried out this reconstruction. The original plan is one that is the most easily worked of any to be found represented in cottage hospital architecture. Everything radiates from a small central inner hall, to the south of which is the chief ward for women, and to the north, that for men. To the west lie the matron's sitting room and the kitchens and new dining room; on the east are placed the operation theatre, a small but excellent section of the hospital where good work is continuously done, together with small wards where private patients are admitted who pay for their residence and also for the doctor's attendance.

The Entrance Hall of the Caterham Cottage Hospital.

The hospital contains seventeen beds and two cots, the greater number of which are constantly occupied. The nursing is done by the matron, Mrs. Grant, who was trained at the London Hospital, with the help of two charge nurses and a probationer. The condition in which we found this hospital on the date of our visit, November 5, 1913, demonstrates the efficiency of the medical and nursing staff and reflects great credit on the matron, who is devoting herself zealously to the best interests of this institution.

The men's ward, facing north, is dependent for its heating upon a central fireplace which consumes an abnormally large amount of coal and gives out a most inadequate amount of heat. This ward must therefore remain unduly cold in the best circumstances. We would recommend the committee to have the inadequate central fireplace removed and a better pattern substituted for it, so as to heat the ward thoroughly and reduce the present consumption of coal.

The hospital has a *post-mortem* room and a rest room, which together form the best mortuary the existing circumstances allow. We hope, however, seeing the many excellencies of the cottage hospital proper, that some man or woman with imagination and heart will make it his or her business to provide that the present mortuary shall be made into a chapel or view room and that a small *post-mortem* room shall be added, as might be done at relatively small cost.

This hospital is exceedingly well kept and maintained. It is one of the most interesting of recent cottage hospitals, and, providing the mortuary is brought up to the high standard of the hospital proper, Caterham must take a leading position among institutions of its type and command the attention of an increasing number of people towards the work it does in the care of the sick. We congratulate the managers, Mrs. Grant, the staff, and all connected with this hospital, upon the high state of efficiency to which their institution has attained.

See a second article on p. 438.

The Newsvendors' Benevolent and Provident Institution.—The Committee of Management of this charitable Society announce that Alderman and Colonel Sir Charles Cheers Wakefield, J.P., has kindly consented to preside at the seventy-fifth annual dinner in aid of the funds of the Newsvendors' Institution on Monday, November 2, 1914, at De Keyser's Royal Hotel.

### THE EPSOM AND EWELL COTTAGE HOSPITAL.

(Second Notice.)

### Are its Committee Asleep?

Our report on this hospital, which was published on June 20 last, has given rise to comment in the local press and to correspondence. "Epsomian" in the *Herald* makes some business-like comments, sounds a note of warning, and urges the committee to institute an independent inquiry. "Asklepios" goes one better. After denying that free medical service is not popular with the medical profession, he proceeds: "It cannot be wondered at that the doctors, seeing that their patients do not always progress as they ought, should desire them to be in the charge of fully qualified nurses with more experience than can be gained in a hospital where the present policy is to keep down the number of cases admitted and to restrict them, as far as possible, to the simplest type and accidents." He then supports "Epsomian's" suggestion by urging the committee not to shirk an inquiry, for that "seems to imply apprehension of the result, which would naturally shake the confidence of the public and have a disastrous effect where a cottage hospital is maintained [as at Epsom] by voluntary contributions."

The support thus given by independent testimony to the suggestions we made is in striking contrast to a letter from Dr. E. C. Daniel, which might fitly be described as an eruption of words from the pen. Dr. Daniel talks of inaccuracies, absurdities, absence of authority and so forth. The one fact proof he relies upon is his statement that in our Report we mistook "the painted ward with three beds for the private ward with one bed." Dr. Daniel's statement in regard to the painted ward is in direct conflict with the statement made during our visit to the hospital. For the rest Dr. Daniel declares the article to be "an attack" upon the medical staff. No person of experience or sound judgment who refers to it will find the Report as it stands to be "an attack" upon anyone. It is merely a practical attempt to move those responsible to stand by the hospital and do their duty. An awakening policy is urgently required to stimulate the managers, to introduce energetic administration, to supply an adequate staff of highly competent nurses, and to cause the committee to meet promptly the just claims of the inhabitants of Epsom to all the hospital care they may desire to have available. The simple truth is that this hospital is capable of doing most excellent work, and that, so far as its accommodation and appliances are concerned, there is no reason why it should not treat with success every class of case within its wards. The inhabitants of Epsom and the local press properly demand that full advantage should be taken of the excellent accommodation this cottage hospital contains, and they properly urge its committee to institute an inquiry, which should be public, so as to satisfy this just demand without further delay.

Unfortunately a short letter from the Honorary Secretary in the local press makes it evident that neither the committee nor himself are aware of their own mistakes and the claim the public have upon them for prompt action. The Honorary Secretary demands "the production of specific cases of complaint, when alone it will be possible for the committee to act." This is merely trifling. We hope there is at least one man of business and energy on this committee who will sweep away evasive nonsense of this kind and insist that a public inquiry shall be held without delay. Such an inquiry should pave the way to the speedy restoration to the people of Epsom of the full usefulness of its Cottage Hospital for the benefit of the people of the district it was established to serve. "Asklepios'" intimation that there is an absence of fully qualified nurses who have had a wide experience in the nursing of all kinds of cases is one which, if it stood alone, would demand the instant setting on foot of the proposed inquiry.

The Epsom and District Medical Society, through its Honorary Secretary, has communicated an official letter to the *Wimbledon Herald* in which they express the opinion that the policy of the Epsom doctors is "worthy of the best history of the profession." It would be strange indeed if they took any other view; but it does not follow that other people will take it too. The editor of this local paper, for one, is obviously against them, for he tells them bluntly that he "would like to see our cottage hospital not less highly popular with the inhabitants of this district than are the hospitals in other districts, but if possible even more popular, though it would be difficult for the residents of Epsom to take a greater pride in their hospital . . ." This disposes effectually of the contentions both of the Epsom and District Medical Society and of the Honorary Secretary of the Epsom and Ewell Cottage Hospital; and we are content to leave the verdict to the inhabitants of the neighbourhood.

Cottingham.—The Hull Corporation Health Committee have resolved to invite tenders on the plans of the city architect for a new sanatorium at Cottingham. The original cost of the hospital was estimated at £17,000, but an amended estimate places the cost at nearly £23,000. This is exclusive of the site on the Cottingham estate.

Hawkmoor.—The Devon County Council Public Health and Housing Committee have approved revised plans for alterations and additions to Hawkmoor Sanatorium at an estimated cost of £15,040.

Saffron Walden.—The Saffron Walden Isolation Hospital Board invite tenders for alterations and additions to the hospital, and for the erection of various buildings at the isolation hospital. Mr. Hugo R. Bird, 10 Union Court, Old Broad Street, E.C., and Brentwood, Essex, is the architect, from whom quantities may be obtained.

## Figures and Tables

**Figure f1:**